# Effects of Design Aesthetics on the Perceived Value of a Product

**DOI:** 10.3389/fpsyg.2021.670800

**Published:** 2021-07-29

**Authors:** Aiqin Shi, Faren Huo, Guanhua Hou

**Affiliations:** ^1^College of Arts and Design, Nanjing Forestry University, Nanjing, China; ^2^Pan Tianshou College of Architecture, Arts and Design, Ningbo University, Ningbo, China

**Keywords:** aesthetic experience, product form, value evaluation, innovation, ERPs

## Abstract

Design aesthetics play a crucial role in product design. Stakeholders expect to develop highly valuable premium products by improving the design aesthetics of products. Nevertheless, the question of how to evaluate the value of design aesthetics has not been fully addressed. In this study, the effects of design aesthetics on the evaluation of the value of a product were investigated through a strictly controlled experiment in which the neural responses of the participants were measured. Forty participants completed the design aesthetics experiment in a laboratory setting. Images of products were divided into two categories: those representing high– and low–design-aesthetic stimuli. Both types of images were labeled with the same price. Overall, the images representing high design aesthetics elicited smaller N100 and lower P200 amplitudes than did the images representing low design aesthetics. This finding indicates that low design aesthetics attracted more attention than high design aesthetics did and that high design aesthetics triggered positive emotions. Low–design-aesthetic products elicited a larger N400 amplitude. This finding reveals the inconsistency between labeled and expected prices. The present study indicates that the N400 component can be used as an indicator for measuring the perceived value of a product in a future product design study. Our study provides event-related potential indicators that can be easily applied in decision making for measuring the perceived value of a product’s design.

## Highlights

- N100 and P200 are two indicators for assessing design aesthetics.
- Smaller N100 and P200 amplitudes indicate a higher level of design aesthetics.
- N400 reflects the value of design aesthetics.
- A larger N400 amplitude suggests that the value of design aesthetics perceived by users is inconsistent with their expectations.

## Introduction

Design aesthetics, which have received increasing attention from researchers in fields, such as ergonomics, neuroscience, and marketing can attract consumers to a product and persuade them to buy it. The aesthetic process is rapid and implicit and can result in sensory pleasure and delight (Goldman, 2001; Handy et al., 2008). Although many studies proved that visual appeal can improve the perceived value of products for consumers (Charters, 2006; Venkatesh and Meamber, 2008; Kristensen et al., 2012; Orth and De Marchi, 2007), little is known regarding how design aesthetics affect the cognitive processing in consumers, especially at the neural level. Without reducing the importance of functionality, brand, and other attributes, the aim of this study was to examine the influence of design aesthetics on the price expectation–based neural response of consumers to products. Specifically, we examined general phases of cognitive neural processing, including early attention processing, affective arousal, and comprehension (Tommaso et al., 2008; Ding et al., 2016). The identified neural mechanism indicates how design aesthetics affect the price expectations of the consumers and has implications for the development of pricing strategies by marketing practitioners.

Aesthetics research has a long history and is interdisciplinary. Philosophers, psychologists, and artists have conducted many studies on aesthetics (Hillyard and Anllo-Vento, 1998). Plato believed that proportion, harmony, and unity constitute the beauty of things (Roggman, 1990). Moreover, Aristotle claimed that the universal elements of beauty are order, symmetry, and definiteness (Roggman, 1990; Hillyard and Anllo-Vento, 1998). Achieving consensus on what is beautiful is difficult because various disciplines provide different perspectives on the nature of aesthetics. A widely accepted definition of aesthetics is “the study of the feelings, concepts, and judgments arising from our appreciation of arts or of the wider class of objects considered moving, or beautiful, or sublime” (Blackburn, 1994; Charters, 2006). Levy et al. (1980) suggested that the core of aesthetics is the “presence or absence of beauty.” Toufani et al. (2017) described design aesthetics as a sensory perception of harmony, beauty, and order. This study adopted the aforementioned definition of design aesthetics.

Aesthetic appreciation involves a shared series of cognitive and evaluation processes that are independent of the aesthetic value of an object. For example, a well-designed product may have aesthetic value, whereas an ordinary product may not; however, both can be appreciated through the same cognitive and evaluative processes (Sibley, 2001; Schepman et al., 2018). According to Zeki (2001), aesthetic appreciation and evaluation are based on neurobiology. Many studies have used positron emission tomography, functional magnetic resonance imaging, and event-related potentials (ERPs) to investigate the neural responses, including visual perception, aesthetic judgment, and preference, induced by appealing, neutral, and ugly pictures (Paradiso et al., 1999; Kawabata and Zeki, 2004; Tommaso et al., 2008). Among the aforementioned techniques, the ERP method has a high temporal resolution and is frequently used to investigate the attention and emotional processes of the brain in aesthetic appreciation (Handy et al., 2008; Wang et al., 2012a; Guo et al., 2018). Therefore, this study investigated the differences in neural response during high– and low–design-aesthetic product perception, which can improve the expected product price.

### Design Aesthetics and Price Expectation

Studies pertaining to design aesthetics suggest that attractive features reside in an object; however, these features are influenced by sociocultural, historical, and technological factors (Frascara and Crozier, 1996). Researchers who considered the perspective of aesthetics as an objective property have developed many aesthetics design rules, such as Gestalt principles, which attribute beauty to symmetry, proximity, similarity, continuance, repetition, and closure (Lewalski, 1988; Baxter, 1995). Currently, people are surrounded by a wide variety of products with similar qualities and functionalities. The aesthetics of a product have a major influence on its first impression on the user (Diefenbach and Hassenzahl, 2011). Users think about a product and its price only when the product arouses their attention or emotion (Norman, 2004). Researchers have confirmed that before obtaining information on the real price of a product, consumers form price expectations according to the visual perception of extrinsic product attributes, such as design aesthetics (Lee and Lou, 1995; Teas and Agarwal, 2000; Jun et al., 2005). Although price expectations and attribute judgments affect the purchase intention of the consumer toward a product (Jun et al., 2005), limited research has focused on measuring how design aesthetics influence the perceived congruency between expected price and real price. This topic is worth exploring because producers spend millions of dollars on the appearance design of products to enhance their product competency (Creusen and Schoormans, 2004). Price plays a crucial role in consumer decision making, and the consistency between the expected and real product prices has a strong influence on product design and sales performance. Visual elements are crucial parts of product design; thus the neural process of design aesthetics should be considered in product design.

The perception of aesthetics follows a hierarchical strategy, which suggests that people classify the aesthetics level of an object through pattern recognition based on visual clues without identifying all the details of the object (Berlyne, 1971). For example, consumers may perceive a product as beautiful or neutral according to their first impression of it. The perception that “beautiful is good” relates beauty to quality. Perceived quality indicates the cognitive evaluation of the intrinsic core benefit sought by consumers from a product (Teas and Agarwal, 2000; Orth and De Marchi, 2007). A beautiful form can improve consumer awareness of the usefulness and usability of a product (Hekkert et al., 2003). However, when the quality of a product is difficult to judge, which occurs frequently, people infer quality or price on the basis of extrinsic cues (Orth and De Marchi, 2007; Mumcu and Kimzan, 2015). Design aesthetics are extrinsic cues that help people evaluate quality and form price expectation, especially for new products or brands that they are unfamiliar with. In the case of unfamiliar products, price expectations are formed according to existing visual cues, such as design aesthetics, without reference to the previous price and brand information. Therefore, in this study, we removed the brand information from products so that the invited participants were unfamiliar with the products.

### Neural Assessment of Aesthetics

Since Zeki (2001) found that neurophysiology can be used to explore the relationship between art and the brain, many researchers have investigated the relationship between aesthetics appreciation and brain function. The studies on design aesthetics that used the positron emission tomography and functional magnetic resonance imaging methods found that the brain regions involved in aesthetics appreciation include the medial orbitofrontal cortex (Paradiso et al., 1999), subcallosal cingulate gyrus, and bilateral insula (Kawabata and Zeki, 2004; Kirk et al., 2009), which are related to reward processing and emotional evaluation. Researchers have used ERPs to measure the visual aesthetics of products and have suggested that the N200, P200, and P300 components are enhanced under affective or intended stimuli (Tommaso et al., 2008; Ding et al., 2016; Guo et al., 2016). ERPs are brain-evoked potentials that reflect specific neural processes under specific stimuli with a high time resolution. The ERP method, which has been used to characterize the time–course mechanism that underlies attention, emotion, and semantic comprehension, was adopted in the current study.

N100 is an attention-related component that has been studied in detail by many researchers (Coull, 1998; Nelson et al., 2015). This component is an early negative ERP component that peaks approximately 100–200 ms after the onset of a stimulus and is distributed in the frontal or parietal lobe. The N100 component distributed in the frontal lobe, which has a latency of 100–150 ms, reflects the influence of a stimulus on visual attention. Moreover, the N100 component distributed in the parietal lobe, which has a latency of 150–200 ms, reflects the influence of a stimulus on identification processing (Hillyard and Anllo-Vento, 1998; Vogel and Luck, 2000). Certain studies have indicated that N100 is mainly affected by physical differences in stimulus materials (Luck et al., 2000; Niu et al., 2008). Therefore, visual attention and identification processing can be affected by factors, such as color, shape, and material. Enhanced N100 amplitudes indicate the commitment of attention resources to extraordinary and meaningful stimuli (Luck et al., 2000). Guo et al. (2018) suggested that small N100 amplitudes may be elicited in the frontal and central brain areas by affective preferences for product appearance. Thus, we assumed that products with low design aesthetics might elicit high N100 amplitudes in the frontal and central lobes.

Aesthetic processing always occurs spontaneously and is a potential source of pleasure (Holbrook and Zirlin, 1983). Images that satisfy affective preferences activate the striatum and parahippocampal region (Yue et al., 2007), which are related to emotional evaluation. P200 is an early positive ERP component that achieves a peak value of approximately 200–300 ms after the onset of stimuli. Moreover, this component is related to emotional arousal. Studies have revealed that an enhanced P200 amplitude is caused by threatening words or images (Carretié et al., 2001a; Correll et al., 2006). Although researchers agree that the P200 amplitude reflects the early automatic emotional processing, no consensus has been reached regarding whether a large or small amplitude of P200 is elicited by high design aesthetics. For example, Wang et al. (2012a) and Ma et al. (2015) found that beautiful pendants elicited smaller P200 amplitudes than less beautiful ones did in an implicit aesthetics experience. However, Herbert et al. (2006) suggested that the P200 amplitude is higher for positive- and negative-valence stimuli than for a neutral-valence baseline. Aesthetics processing involves attention and the experience of emotions; therefore, another objective of this study was to investigate whether large or small P200 amplitudes are elicited by a product with high design aesthetics.

N400 is a negative ERP component associated with comprehension. The peak N400 amplitude occurs approximately 300–500 ms after the onset of stimuli. N400 is sensitive to semantic incongruence and has been observed in many studies on semantic conflict related to non-verbal stimuli that used elements, such as pictures, traffic signs, and mathematic symbols (Barrett and Rugg, 1990; Hou and Lu, 2018). Studies have indicated that large N400 amplitudes can be elicited using expressions with semantic incongruence. For instance, a larger N400 amplitude was elicited when animal names were used with vehicle pictures than when appropriate vehicle names were used (Ma et al., 2016). Similarly, semantically incongruent traffic sign–word pairs caused high N400 amplitudes (Hou and Lu, 2018). Overall, the N400 amplitude is a suitable indicator for detecting semantic incongruence or conflict regardless of the stimulus modality. On the basis of the aforementioned findings, the current study assumed that a relatively large N400 amplitude is elicited when labeled prices are inconsistent with expected prices.

In this study, we investigated the effects of design aesthetics on the value evaluation of a product by using the ERP method. We assumed that products with high design aesthetics would attract more attention and elicit a larger N100 amplitude than would products with low design aesthetics. Moreover, products with high design aesthetics were assumed to induce positive amplitude values for the P200 component. Most importantly, we hypothesize that design aesthetics add value to a product. The pairing of a low–design-aesthetic product with a high price may lead to cognitive incongruences (Lu and Hou, 2020). This hypothesis was tested in the current study by the large N400 values elicited by products with low design aesthetics that were labeled with a relatively high price.

## Materials and Methods

### Participants

In this study, 40 participants were recruited from Ningbo University. Data pertaining to two participants were excluded because of excessive recording artifacts. The remaining participants (19 men and 19 women; age: 22 ± 1.09 years) had normal or correct-to-normal visual acuity and no history of mental illness. All the participants were right-handed according to a revised version of the handedness questionnaire of Oldfield (1971). Moreover, the participants had different majors and did not have any aesthetics or design education background. All of them signed informed consent forms and were paid CN¥100 for participating in the experiment. The Internal Review Board of the Neural Ergonomics Lab approved the current study.

### Experimental Stimuli

The Red Dot Design Award is an international product design and communication design prize awarded by the Design Zentrum Nordrhein Westfalen, Germany. For this award, well-designed products are selected by competent expert juries in the areas of product design, communication design, and design concepts. The aforementioned juries not only consider aesthetic quality but also the creativity, utility, and manufacturability of a product. Products that have received the Red Dot Design Award should be suitable stimuli for testing our hypothesis because these products have been filtered on the basis of identical authority evaluation and can be deemed to be more beautiful than low–design-aesthetic products. The pictures of 60 products with high design aesthetics were selected from the website of the Red Dot Design Award as stimuli in this study. Moreover, the pictures of 60 products with low design aesthetics were obtained from online shopping companies, such as Amazon. To avoid the influence of function on the product value, products (i.e., electronic tools) with similar functions were selected. All the product pictures were preprocessed using Adobe Photoshop CS (Adobe, CA, United States) and were converted to black-and-white images with the same luminance, shade, and size.

**FIGURE 1:**
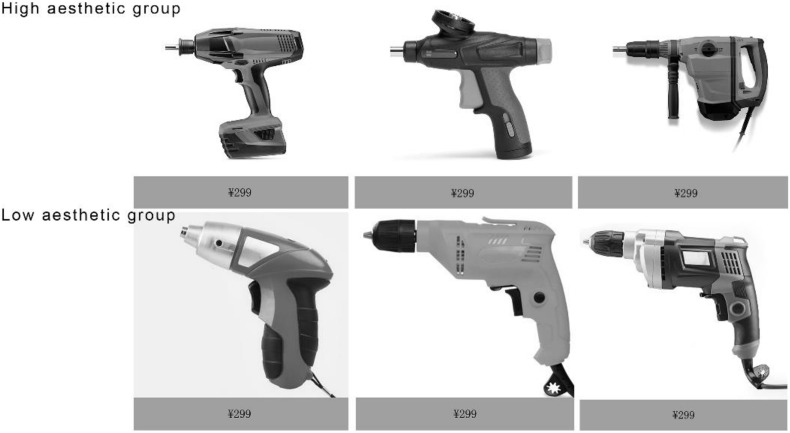
Examples of the experimental stimuli.

Twenty volunteers who had not participated in the ERP experiment rated the design aesthetics of the selected products in a pilot study on a 7-point Likert scale ranging from 1 for very low design aesthetics to 7 for very high design aesthetics. A total of 60 products were selected as experimental stimuli. Thirty top-ranking products with high design aesthetics were defined as high–design-aesthetic products, and 30 bottom-ranking products with low design aesthetics were defined as low–design-aesthetic products. The mean rating scores were significantly different between the aforementioned two product groups (*t* = 11.32, *p* = 0.001, Cohen’s *D* = 5.19). These scores were examined through a paired *t*-test. Figure 1 displays some examples of the experimental stimuli.

All the product pictures were presented with the same price label of CN¥299 (approximately US$46.45) and no brand information. The displayed price was relatively high. According to our survey, most electric drills produced by not so famous brands cost approximately CN¥100–200 (approximately US$14.2–28.4). Drills from Bosch, which is a well-known electrical tool factory, cost approximately CN¥260–290. Therefore, we expected the participants to react differently to the combination of different aesthetics and the high price.

### Experimental Procedure

**FIGURE 2:**
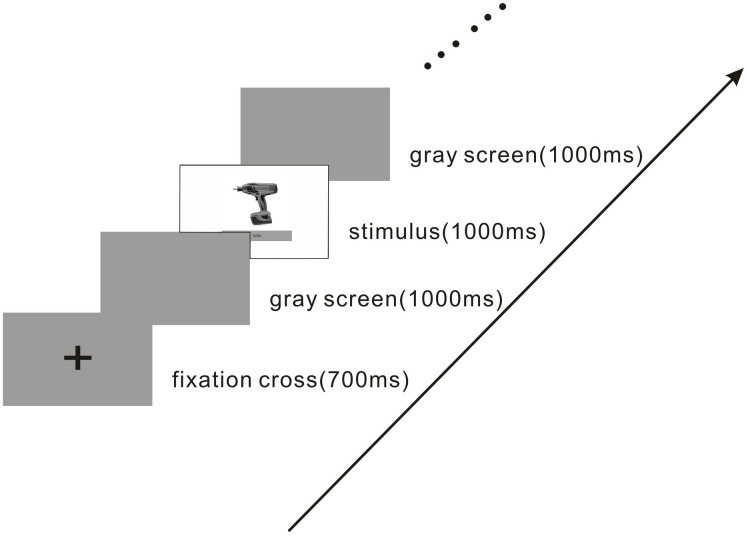
Experimental procedure. In each trial, the participants viewed a product picture with a fixed price for 1,000 ms and then determined whether they would buy the product. The participants were asked to make a decision as quickly as possible.

Both groups of stimuli were randomly presented twice to the participants through E-prime 2.0 software, which assigned an equal probability to both the groups. The participants were asked to face a computer screen and sit comfortably at a distance of 70 cm from the screen in a soundproof laboratory with a visual angle of 2.58° × 2.4°. The experimental procedure comprised two groups of 60 trials each. The stimuli were displayed at the center of the screen with a gray background. The experimental procedure is illustrated in Figure 2. In each trial, a “+” sign was first presented for 700 ms. Next, a gray screen was displayed for 1,000 ms, after which a picture of a product with a fixed price was displayed for 1,000 ms. During the presentation, the participants were asked to judge within 1,500 ms whether they would like to buy the displayed product. Finger assignments were counterbalanced across the participants. Half of the participants were asked to press “2” for “intend to buy” and “3” for “do not intend to buy.” The other half were asked to press “3” for “intend to buy” and “2” for “do not intend to buy” to ensure counterbalance. Finally, another gray screen was presented for 1,000 ms before the next trial. The participants completed a brief practice exercise before the experiment.

### Data Acquisition and Analysis

A Neuroscan Synamp2 Amplifier equipped with an electrode cap containing 64 Ag/AgCl electrodes was used to record electroencephalogram (EEG) data continuously according to the standard international 10–20 system. The passband was 0.05–50 Hz, and the sampling rate was 1,000 Hz. The left and right mastoids served as locations of the reference electrodes, and an FCz electrode was used as the ground electrode. The electrode impedance was maintained below 5 kΩ in the experiment.

**FIGURE 3:**
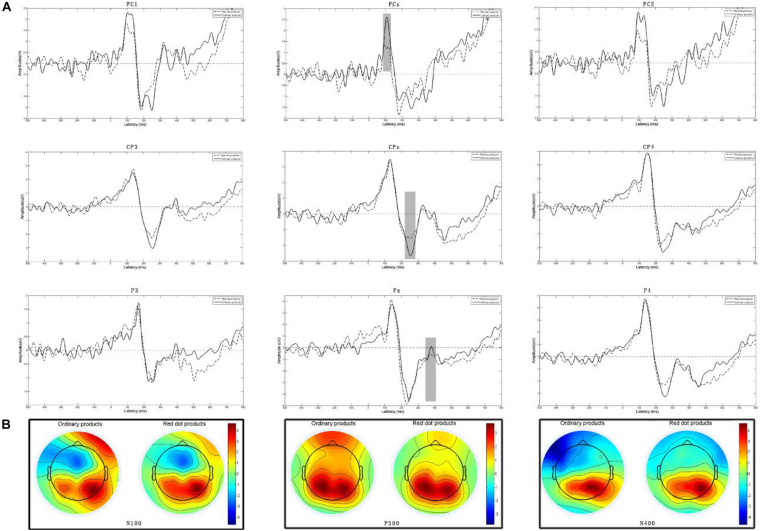
**(A)** Grand-average ERPs elicited by high– and low–design-aesthetic products at nine electrodes in the frontal, central, and parietal regions of the brain. **(B)** Topographic maps of N100 (90–110 ms), P200 (240–260 ms), and N400 (370–390 ms).

We used the MATLAB 2013b EEGlab toolbox (MathWorks, MA, United States) to preprocess the EEG data offline. The EEG signals were evaluated with reference to the average of the left and right mastoids and digitally filtered through a low-pass filter with a cutoff frequency between 0.1 and 30 Hz. The EEG recordings were segmented from 200 ms before stimulus onset to 800 ms after stimulus onset, with the data for the first 200 ms being used as the baseline data. Low signal-to-noise ratio segments were rejected, and independent component analysis was conducted to eliminate EEG artifacts. After the artifacts had been eliminated, 52.3 [standard deviation (SD) = 4.1] trials remained for the Red Dot Award condition and 47.6 (*SD* = 6.2) trials remained for the ordinary product condition. Finally, the EEG data were averaged separately for the products that received the Red Dot Award and the low–design-aesthetic products. The grand-average ERP waveforms and topographic maps are illustrated in Figure 3. We performed repeated measures analysis of variance (ANOVA) of the ERPs by using SPSS 19.0 software (IBM, NY, United States). Bonferroni’s method was adopted for multiple comparison correction when appropriate. Behavioral data (response time under different conditions) were analyzed through paired *t*-tests.

## Results

### Behavioral Results

The behavioral data were divided into four conditions for each participant: intend to buy a high–design-aesthetic product, do not intend to buy a high–design-aesthetic product, intend to buy a low–design-aesthetic product, and do not intend to buy a low–design-aesthetic product. Before conducting statistical analysis, we examined the data distribution by using the Shapiro–Wilk test. The results of this test indicated that the *p* values of response time for the four conditions were above 0.05, indicating that the data had a normal distribution. Repeated measures ANOVA was performed to analyze the behavioral data. We performed a verification of the sphericity hypothesis, and the results indicated that *p* = 0.32, which conforms to the conditions for performing repeated measures ANOVA. The statistical results revealed that design aesthetics significantly influenced the response time (*F* = 65.28, *p* < 0.001, η^2^*_p_* = 0.55). The participants exhibited a longer mean response time during the decision-making process for high–design-aesthetic products (mean ± SD = 731.14 ± 74.38) than for low–design-aesthetic products (mean ± SD = 701.58 ± 69.47). In addition, purchasing intention significantly influenced the participants’ response time (*F* = 31.72, *p* < 0.03, η^2^*_p_* = 0.37). The results revealed that the participants who did not intend to buy a product exhibited a shorter response time (mean ± SD = 706.35 ± 79.17) than did those who intended to buy a product (mean ± SD = 738.51 ± 68.83). The interaction between design aesthetics and purchasing intention was not significant (*F* = 2.87, *p* = 0.163, η^2^*_p_* = 0.13). Furthermore, the mean ratios of the intent to buy high— and low–design-aesthetic products were determined to be 87.6 ± 13.4% and 67.3 ± 10.5%, respectively. The intent-to-buy ratio was calculated as the number of “intend-to-buy” reactions divided by the total number of reactions. The statistical analysis results revealed a significant difference between the high— and low–aesthetic design products (*t* = 7.58, *p* < 0.001, Cohen’s *D* = 1.70).

### ERP Results

The ERP waveforms and topographic maps displayed in Figure 3 indicate the presence of two negative components with latencies of 80–120 and 370–410 ms in the frontal and parietal area, respectively. Studies have indicated that these components correspond to N100 and N400 (Barrett and Rugg, 1990; Coull, 1998). Moreover, a positive component with a latency of 220–260 ms was observed in the parietal region of the brain. This component corresponds to P200, as indicated by studies on emotional arousal (Wang et al., 2012a; Ma et al., 2015). Thus, a series of within-group repeated measures ANOVAs were conducted on the N100, P200, and N400 amplitudes in the ERP analysis. Because attentional N100 often exhibits an anterior distribution (Coull, 1998), frontal and parietal P200 activity was elicited during the processing of affective pictures (Yuan et al., 2007; Ma et al., 2015). We selected the FC1, FCz, FC2, F1, Fz, and F2 electrodes to analyze N100 and selected the FC1, FCz, FC2, CP3, CPz, and CP4 electrodes to analyze P200. Many studies have explored the semantic processing of picture–word pairs and discovered that N400 activity is elicited and distributed in the parietal regions of the brain (Ma et al., 2016; Hou and Lu, 2018). We selected the CP3, CPz, CP2, P–P3, Pz, and P4 electrodes to analyze N400. First, a 2 (design aesthetics: high design aesthetics vs. low design aesthetics) × 2 (localization: FC–FC1, FCz, and FC2 vs. F–F1, Fz, and F2) × 3 (lateralization—left: FC1 and F1, middle: FCz and Fz, and right: FC2 and F2) within-group repeated measures ANOVA was conducted on the mean N100 amplitudes. Next, a 2 (design aesthetics: high design aesthetics vs. low design aesthetics) × 2 (localization: FC–FC1, FCz, and FC2 vs. CP–CP3, CPz, and CP2) × 3 (lateralization—left: CP3 and P3, middle: CPz and Pz, and right: CP2 and P2) within-participant repeated measures ANOVA was performed on the mean P200 amplitudes. Finally, a 2 (design aesthetics: high design aesthetics vs. low design aesthetics) × 2 (localization: CP–CP3, CPz, and CP2 vs. P–P3, Pz, and P4) × 3 (lateralization—left: CP3 and P3, middle: CPz and Pz, and right: CP2 and P2) within-participant repeated measures ANOVA was performed on the mean N400 amplitudes.

The peak N100, P200, and N400 amplitudes were analyzed in this study. The mean N100, P200, and N400 amplitudes were calculated for time windows of 90–110, 240–260, and 370–390 ms after the onset of a stimulus, respectively. The ANOVA results for the N100 amplitude revealed that the main effects of design aesthetics [*F*_(__1_,_37__)_ = 8.801, *p* = 0.009,η^2^ = 0.251] and lateralization [*F*_(__2_,_74__)_ = 8.177, *p* = 0.014, η^2^ = 0.012] were significant. However, the main effect of localization [*F*_(__1_,_37__)_ = 0.316, *p* = 0.582, η^2^ = 0.002] was not significant. No significant interactions were noted among the aforementioned factors. The average N100 amplitude for the low–design-aesthetic products (mean ± SD = −1.396 ± 0.625) was more negative than that for the products that won the Red Dot Award (mean ± SD = −0.778 ± 0.612). With regard to the lateralization effect, significant differences were observed between the middle and right lines (mean_middle_ = −1.632, mean_left_ = −1.183, *p* = 0.034) and between the middle and left lines (mean_middle_ = −1.632, mean_*right*_ = −1.097, *p* = 0.019); however, the difference between the left and right lines (mean_left_ = −1.183, mean_right_ = −1.097, *p* = 0.35) was not significant.

The ANOVA results obtained for the P200 amplitude indicated that the main effects of design aesthetics [*F*_(__1_,_37__)_ = 9.790, *p* = 0.007, η^2^ = 0.379] and localization [*F*_(__1_,_37__)_ = 5.482, *p* = 0.032, η^2^ = 0.218] were significant. However, the main effect of lateralization [*F*_(__2_,_74__)_ < 1, *p* = 0.523, η^2^ = 0.035] was not significant. No significant interactions were observed among the aforementioned factors. The P200 amplitude (i.e., positive amplitude) elicited for the high–design-aesthetic products (mean ± SD = 1.968 ± 0.557) was larger than that elicited for the low–design-aesthetic group (mean ± SD = 1.357 ± 0.814) at the frontal lobe; however, the difference was not significant (*p* = 0.082). By contrast, the P200 amplitude (i.e., positive amplitude) elicited for the high–design-aesthetic products (mean ± SD = 1.697 ± 0.337) was significantly smaller than that elicited for the low–design-aesthetic products (mean ± SD = 2.571 ± 0.814) at the parietal lobe.

The ANOVA analysis results obtained for the N400 amplitude indicated that the main effects of design aesthetics [*F*_(__1_,_37__)_ = 5.837, *p* = 0.011, η^2^ = 0.163] and lateralization [*F*_(__2_,_74__)_ = 8.217, *p* = 0.007, η^2^ = 0.332] were significant. However, the main effect of localization [*F*_(__1_,_37__)_ = 1.660, *p* = 0.011, η^2^ = 0.074] was not significant. No significant interactions were observed among the aforementioned factors. The average N400 amplitude (mean ± SD = −0.245 ± 0.310) elicited for the low–design-aesthetic products was more negative than that elicited for the high–design-aesthetic products (mean ± SD = 0.29 ± 0.457). A pairwise comparison of the lateralization effect revealed a significant difference between the left and right lines (mean_left_ = −0.276, mean_right_ = 0.793, *p* = 0.01).

## Discussion

This study aimed to determine the effects of design aesthetics on product value evaluation through the ERP method. Specifically, the effects of design aesthetics on individuals’ attention, emotions, and product value evaluation were determined through measurement of the N100, P200, and N400 amplitudes. First, the behavioral results revealed that design aesthetics had a significant influence on the purchase intentions of the participants. The buying intent ratio for high–design-aesthetic products was significantly higher than that for low–design-aesthetic products. Second, the N100 amplitudes in the frontal region of the brain indicated that the high–design-aesthetic products attracted more attention from the participants than did the low–design-aesthetic products. Moreover, the P200 amplitude in the central region of the brain suggested that the high–design-aesthetic products induced more positive emotions than did the low–design-aesthetic products. Third, the results of this study revealed that the N400 amplitude can indicate not only semantic incongruence but also the inconsistency between expected and labeled product prices. In this study, larger N400 amplitudes revealed greater inconsistencies between the expected product price based on design aesthetics and the real product price. The ERP results provided neural evidence for the enhancement effect of high design aesthetics on the product value.

### Effects of Design Aesthetics on the Purchase Intention and Behavior of the Participants

As expected, in general, design aesthetics significantly influenced consumer behavior in this study, which is in line with the results of previous studies (Cai and Xu, 2011; Hagtvedt and Patrick, 2014). In particular, the current study revealed that the buying intent ratio was significantly higher for the high–design-aesthetic products than for the low–design-aesthetic products. Moreover, the low–design-aesthetic products had a significantly lower response time for rejection than did the high–design-aesthetic products.

The results of this study revealed that the buying intent ratios for high–design-aesthetic products were significantly higher than those for the low–design-aesthetic products (87.6 ± 13.4% vs. 67.3 ± 10.5%, *t* = 7.58, *p* < 0.001). This finding indicates that design aesthetics significantly influenced the purchase decisions of the participants. Our findings can be partially explained by the findings of Hagtvedt and Patrick (2014). They investigated the relationship between design aesthetics and functionality as well as the influence of these parameters on consumer behavior. They found that design aesthetics play a crucial role in the purchase behaviors of the consumers, especially when functional demands are met. Moreover, design aesthetics can compensate for minor flaws in functionality This finding indicates that consumers prefer appealing products and explains why high design aesthetics can improve the buying intent of consumers.

We also measured the response time (time required to decide whether to buy a product) when the participants viewed pictures of high– and low–design-aesthetic products. Behavioral data grouped according to the response time taken by the participants indicated that they spent a significantly shorter time deciding to not buy a product than deciding to buy a product, which indicates that more cognitive resources were allocated to the decision to buy. Guo et al. (2018) provided one explanation for such a result. They investigated design aesthetics on the basis of the preferences of the people and found that participants responded more quickly to the products that they disliked. Ma et al. (2016) provided another explanation for the aforementioned result. They asked participants to judge whether the semantics of a picture–word pair were congruent. The results reported by Ma et al. (2016) revealed that the response times of the participants for incongruent product–word pairs were significantly shorter than those for congruent pairs. The stimuli in the current study were pictures of products with their price. Low design aesthetics paired with a high price may have been inconsistent with the expectations of the participants; thus, a shorter response time was observed for low–design-aesthetic products than for high–design-aesthetic products. The aforementioned result indicates that design aesthetics significantly affected the purchase intention of the participants and the response time taken for decision making by the participants.

### Effects of Design Aesthetics on the Perceptions and Emotions of the Users

The significant differences in the ERP data between high– and low–design-aesthetic products with the same price suggest that people evaluate products according to special aesthetic experiences, including attention perception and emotional arousal. The beautiful products, which were awarded the Red Dot Design Award, elicited more attention in the early processing stage with a smaller N100 amplitude than did the low–design-aesthetic products. The arousal of positive emotions in the early stage was reflected by the P200 amplitude, which was smaller for the high–design-aesthetic products than for the low–design-aesthetic products.

N100 is an early rapid automatic neural activity component that cannot be controlled by individuals’ cognition. The distribution of N100 in the frontal area of the brain reflects the allocation of attentional resources (Righi et al., 2014). Moreover, a higher N100 amplitude suggests the allocation of more attention resources (Vogel and Luck, 2000). In intentional discrimination tasks conducted in a previous study, N100 was distributed in the frontal and central areas of the brain, reflected a discrimination process, and exhibited significant differences between affective and neutral stimuli (Keil et al., 2001). Jacobsen and Höfel (2003) observed an enhanced amplitude of early frontal negativity for judgments of not so beautiful objects. They also suggested that the judgment of beauty involves a two-stage process consisting of early anterior front median impression formation and aesthetic-specific evaluative categorization. In the current study, a significantly larger N100 amplitude was elicited in the frontal area, especially at the FCz electrode (Figure 3A), for the low–design-aesthetic products than for the high–design-aesthetic products. A likely explanation for this phenomenon is that the participants automatically identified the product appearance when they formed their purchase intention. Our results are consistent with those obtained by Guo et al. (2018), who used pictures of humidifiers as stimuli and found that larger N100 amplitudes were elicited for neutral humidifier pictures than for liked or disliked humidifier pictures. Moreover, Ding et al. (2016) used ERPs to investigate the visual aesthetic perception of smart phone appearance and found that neutral pictures elicited larger N100 amplitudes than did liked or disliked pictures. As indicated by previous studies, this result suggests that more attentional resources are allocated to low–design-aesthetic products than to high–design-aesthetic products.

Studies have revealed that emotional processing occurs in the early stage of aesthetic experience (Tommaso et al., 2008; Wang et al., 2012a; Guo et al., 2016). Moreover, P200 is sensitive to emotional stimuli (Tortosa et al., 2013). The results obtained for the P200 amplitude in this study are consistent with those obtained by Tortosa et al. (2013), which indicated that high– and low–design-aesthetic products elicit different P200 amplitudes. However, contradictory results have been obtained in the relevant literature for P200. Many studies have found that relatively small P200 amplitudes are elicited for beautiful artifacts or positive emotional stimuli (Wang et al., 2012a; Tortosa et al., 2013; Ma et al., 2015); however, some studies have suggested that relatively large P200 amplitudes can be elicited by positive and negative emotional stimuli (Correll et al., 2006; Herbert et al., 2006). In this study, the P200 amplitude in the parietal region of the brain was significantly higher for the high–design-aesthetic products than for the low–design-aesthetic products. This result is consistent with those obtained in previous studies (Carretié et al., 2001b; Wang et al., 2012a; Tortosa et al., 2013; Ma et al., 2015; Liu et al., 2019). A functional magnetic resonance imaging study indicated that the medial orbitofrontal cortex and subcallosal cingulate gyrus are activated when an individual makes an aesthetic judgment (Kirk et al., 2009). These brain regions are associated with reward processing and emotional arousal. Our results suggest that design-aesthetic experience involves emotional processing in the early stage of aesthetic evaluation and that high design aesthetics arouse positive emotions with a relatively low amplitude for P200.

However, the P200 amplitudes in the frontal region were inconsistent with those in the parietal region in this study. The P200 amplitudes in the frontal area were larger for the high–design-aesthetic products than for the low–design-aesthetic products; however, the difference was not significant. Studies have suggested that the P200 amplitude in the frontal region reflects the detection and analysis of task-relevant features (Luck and Hillyard, 1994). Ma et al. (2001) found that a larger P200 amplitude is elicited in the frontal region by beautiful products than by not so beautiful products. A likely explanation for this result is that design aesthetics are associated with shape, size, or orientation, which are sensitive to the P200 amplitude in the frontal area. Thus, we attribute the larger P200 amplitude elicited by the high–design-aesthetic products to their design features.

### Effects of Design Aesthetics on the Perceived Value of a Product

Kutas and Hillyard (1980) were the first to reveal that the N400 amplitude can act as an index of semantic processing, and numerous studies proved that N400 is sensitive to semantic incongruence and semantic ambiguity (Kutas and Hillyard, 1980; Hamm et al., 2002). Early studies that adopted the ERP method used the N400 amplitude mainly for examining language and text comprehension (Kutas and Hillyard, 1980). Many semantic studies on topics, such as spoken language and body language have found that N400 is sensitive to semantic incongruence (Kutas and Hillyard, 1980; Balconi and Amenta, 2010; Kutas and Federmeier, 2011). Multimodal metaphors are an extended application of N400 for semantic studies. These metaphors are constructed in the target and source domains displayed in different types of representations, such as word–word and picture–word combinations (Forceville and Urios-Aparisi, 2009; Schneider et al., 2014; Ma et al., 2016). In the current study, the source domain was the displayed product price and the target domains were the displayed electrical tools. Metaphor comprehension is an analogical mapping process that involves the retrieval of specific features of the source domain from memory before mapping. Pictorial metaphors are considerably easier to comprehend than verbal metaphors are because the features of pictorial metaphors can be visualized more easily (Forceville and Urios-Aparisi, 2009). Therefore, the current study used price as a metaphor for the value of design aesthetics.

Studies have revealed that N400 can be used to indicate inconsistency with expectations in product or sign (Wang et al., 2012b; Ma et al., 2016). The current study used the combination of a picture of an electric tool and a written price to explore the cognitive process of value evaluation through the measurement of ERPs. The results indicated that a significantly larger N400 amplitude was elicited for pictures of low–design-aesthetic products than for pictures of high–design-aesthetic products, which indicates that a high N400 amplitude was elicited when the labeled price was inconsistent with expectations. Inconsistent picture–price combinations elicited larger N400 amplitudes than did a consistent picture–price combinations (Ma et al., 2016; Lu and Hou, 2020). Wang et al. (2012b) used N400 as an indicator to detect brand inconsistency with product attributes. They suggested that N400 reflects a crucial unconscious categorization in the selection of branded products. N400 has been reported to be mainly distributed in the frontal regions in studies using word–word combinations (Hamm et al., 2002; Schneider et al., 2014) and in the parietal and occipital regions in studies using picture–word combinations (Ma et al., 2016; Lu and Hou, 2020). In the current study, N400 was distributed in the occipital regions, which is consistent with the results of previous studies (Ma et al., 2016). A possible explanation for this result is that pictorial information must be preprocessed and integrated into the relevant context during the examination of pictorial metaphors.

Louis Sullivan claimed that “the form follows the function.” The separation of function and design aesthetics is impractical. Although we attempted to control for function in the current study, the participants associated design aesthetics with different functions. An inconsistent expectation between design aesthetics and price has two aspects. First, high design aesthetics indicate high functionality. A study revealed that design aesthetics can compensate for some functional defects (Hagtvedt and Patrick, 2014). High design aesthetics increase people’s trust in a product and increase the value of the product. Second, design aesthetics is a potential source of pleasure that increases the product value. Studies have reported that product aesthetics have a significant influence on the preferences of the consumers (Stanton et al., 2016). The aesthetic response of the consumers positively influences their judgments regarding a product. Therefore, high design aesthetics increase the product value, whereas low design aesthetics cause consumers to perceive a product as not being worth its listed price.

Overall, the current study revealed that N400 can reflect the inconsistency between design aesthetics and price and demonstrated that design aesthetics significantly influence the product value perceived by individuals. The ERP results suggest that an excellent design increases the product value.

### Theoretical and Managerial Implications

This study provides valuable information on the application of N400. Studies have indicated that presenting people with verbal stimuli that they perceive as exhibiting semantic conflict can elicit N400 (Barrett and Rugg, 1990). N400 has been found to be effective for detecting semantic conflict in non-verbal stimuli (Ma et al., 2016; Lu and Hou, 2020). The current study found that N300 and N400 can be used to detect the expected consistency between products and their prices. This study enhances the theoretical understanding of N400.

From a management perspective, this study demonstrates the value of a suitable product design. Product aesthetics play a crucial role in new product development. Design aesthetics significantly influence individuals’ responses, including their purchase intention, attention distribution, and emotions. Moreover, an appealing design increases the product value. Product developers should understand the importance of product design and use the ERP method to assist their decision making regarding product design.

## Limitations and Conclusion

This study used the ERP method to investigate the effects of design aesthetics on the perceived value of a product. The ERP method reveals the cognitive mechanism of aesthetics processing. However, design aesthetics have many attributes, such as order, symmetry, and complexity, which were not strictly controlled in this study for ecological validity. The effects of these attributes on the aesthetics-related responses of individuals are worthy of investigation. Moreover, each stimulus was presented twice randomly to each participant, which may have influenced their behavioral responses.

With regard to the experimental design, we controlled for irrelevant variables by removing brand information and selecting electronic tools with similar functionality. The same price was then set for products with high design aesthetics and low design aesthetics to investigate the effects of design aesthetics on the perceived product value. We recruited participants who did not have any experience in using electronic tools to ensure that the responses by the participants were based only on design aesthetics. Thus, future studies should investigate whether our results are valid for different products and for participants with experience using the investigated products.

The current study measured N100, P200, and N400 amplitudes to determine the influence of design aesthetics on the perceived value of a product. Low–design-aesthetic products attracted higher attention than did high–design-aesthetic products, as reflected by the larger N100 amplitudes for the low–design-aesthetic products. Moreover, high–design-aesthetic products induced positive emotions, as indicated by low P200 amplitudes. N400 implicitly reflected the perceived product value. In conclusion, this study presents ERP results for the cognitive processing of aesthetics and explains why high design aesthetics improve product value from a neural science perspective.

## Data Availability Statement

The raw data supporting the conclusions of this article will be made available by the authors, without undue reservation.

## Ethics Statement

The studies involving human participants were reviewed and approved by Inclusive User Experience Design Lab. The patients/participants provided their written informed consent to participate in this study.

## Author Contributions

AS defined the research gap and prepared the draft of this study. FH designed and organized the experiment. GH processed the data and supervised the writing of this Study. All authors contributed to the article and approved the submitted version.

## Conflict of Interest

The authors declare that the research was conducted in the absence of any commercial or financial relationships that could be construed as a potential conflict of interest.

## Publisher’s Note

All claims expressed in this article are solely those of the authors and do not necessarily represent those of their affiliated organizations, or those of the publisher, the editors and the reviewers. Any product that may be evaluated in this article, or claim that may be made by its manufacturer, is not guaranteed or endorsed by the publisher.
